# Functional and Structural Outcomes of Photodynamic Therapy (PDT) With or Without Eplerenone for Central Serous Chorioretinopathy

**DOI:** 10.3390/jcm15145701

**Published:** 2026-07-21

**Authors:** Giulia Gregori, Chiara Centini, Lorenzo Mangoni, Alessio Muzi, Daniela Fruttini, Alberto Quarta, Maria Ludovica Ruggeri, Clara Rizzo, Giacomo Bongiovanni, Rodolfo Mastropasqua, Cesare Mariotti, Marco Lupidi

**Affiliations:** 1 Eye Clinic, Department of Experimental and Clinical Medicine, Polytechnic University of Marche, 60121 Ancona, Italy; g.gregori98@gmail.com (G.G.); cent.chiara@gmail.com (C.C.); lollomangoni92@gmail.com (L.M.); giacomobongiovanni92@gmail.com (G.B.); c.mariotti@univpm.it (C.M.); 2 Eye Clinic, Humanitas Gradenigo, 10153 Turin, Italy; alessiomuzi90@gmail.com; 3 Department of Medicine and Surgery, University of Perugia, S. Maria della Misericordia Hospital, 06129 Perugia, Italy; daniela.fruttini@unipg.it; 4 Department of Neuroscience, Imaging and Clinical Sciences, University G. D’Annunzio Chieti-Pescara, 66013 Chieti, Italy; alberto.quarta.96@gmail.com (A.Q.); rodolfo.mastropasqua@gmail.com (R.M.); 5 Department of Neurosciences, Psychology, Drug Research, and Child Health, Eye Clinic, University of Florence, AOU Careggi, 50139 Florence, Italy; clararizzo2@gmail.com; 6 Fondazione Italiana Macula ETS, Dipartimento di Neuroscienze, Riabilitazione, Oftalmologia, Genetica e Scienze Materno-Infantili (DINOGMI), University Eye Clinic, 16132 Genova, Italy

**Keywords:** central serous chorioretinopathy, photodynamic therapy, eplerenone, artificial intelligence, subretinal fluid, optical coherence tomography

## Abstract

**Background/Objectives**: To evaluate whether the combination of oral eplerenone and half-dose full-fluence photodynamic therapy (HD-FF PDT) provides greater efficacy than HD-FF PDT alone in persistent central serous chorioretinopathy (CSCR). **Methods**: This monocentric, retrospective, observational study included patients with persistent (>6 months) simple or complex CSCR who had previously undergone either half-dose full-fluence photodynamic therapy (HD-FF PDT) alone or HD-FF PDT combined with oral eplerenone as part of routine clinical practice between September 2024 and March 2025. Functional and morphological data collected at baseline and at 1, 3, and 6 months after treatment were retrospectively reviewed. An artificial intelligence–based algorithm was used to analyze OCT scans, quantifying subretinal and intraretinal fluid volumes (SRFV, IRFV) and assessing ellipsoid zone and external limiting membrane integrity, hyperreflective foci, subfoveal choroidal thickness (SCT), and central macular thickness (CMT). **Results**: Fifty patients (53 eyes; mean age 52 years) were included, with no significant baseline differences between groups. Best-corrected visual acuity improved more significantly in Group B at 1 and 6 months (*p* = 0.032 and *p* = 0.009, respectively). At 6 months, subretinal fluid volume (SRFV), quantified by AI-based OCT analysis, was significantly lower in the combined therapy group (*p* = 0.014). An AI-defined complete resolution of subretinal fluid (SRFV < 0.010 mm^3^) was achieved more frequently in Group B than in Group A (77% vs. 22%, *p* = 0.001). **Conclusions**: Although HD-FF PDT remains the standard treatment for persistent CSCR, adjunctive therapy with the mineralocorticoid receptor antagonist eplerenone may enhance subretinal fluid reabsorption and improve mid-term anatomical and functional outcomes.

## 1. Introduction

Central Serous Chorioretinopathy (CSCR) is a prevalent retinal disorder first described by Albrecht von Graefe in 1866. It is characterized by idiopathic focal serous detachment of the neural retina and/or retinal pigment epithelium (RPE) at the posterior pole, resulting from leakage in the choriocapillaris. Symptoms of CSCR typically include impaired and/or distorted central vision together with altered color perception, and the disease is often associated with reduced vision-related quality of life [1,2,3,4]. CSCR is recognized as the fourth leading cause of vision-threatening non-surgical retinal diseases, following age-related macular degeneration (AMD), diabetic retinopathy (DR), and retinal vein occlusion (RVO). It most frequently affects individuals aged between 39 and 51 years, with an estimated incidence of approximately 9.9 per 100,000 in men and 1.7 per 100,000 in women [5,6].

CSCR is the first condition described in the pachychoroid disease spectrum, a relatively recent term introduced to denote a thickened Haller choroidal layer with attenuation of the Sattler and choriocapillaris layers, sometimes accompanied by RPE abnormalities overlying the pachyvessels [7].

The pathophysiology of CSCR involves choroidal hyperpermeability and dysfunction of the RPE. Conventionally, the related risk factors are stress, corticosteroid use, type-A personality, obstructive sleep apnea, pregnancy, and vigorous physical activity [8,9,10].

Traditionally, CSCR is classified into acute and chronic [11]. The CSCR International Group tried to validate a new classification system to better define distinct disease patterns and prognostic biomarkers. This novel system divides CSCR into “simple” and “complex” based on the extension of RPE alterations and other outer retinal changes in order to rectify the discrepancies in previous classification (acute/chronic) [12].

Choosing the best treatment for CSCR can be challenging due to the significant variability in disease presentation and clinical course, the current lack of consensus regarding a classification system, and the poorly understood pathophysiology of the disease [13,14,15]. The goal is to achieve a complete resolution of the SRF, in order to preserve the outer neurosensory retinal layers and to prevent irreversible damage which even a small amount of persistent SRF may cause [16].

CSCR treatment’s options are several, such as photodynamic therapy (PDT), conventional focal laser photocoagulation, subthreshold micropulse laser (SML), intravitreal injections of anti-vascular endothelial growth factor (VEGF) compounds and oral eplerenone or spironolactone. PDT has been adapted from its oncological origins to address CSCR. It employs verteporfin, a photosensitizing agent which accumulates in abnormal choroidal vessels. When exposed to a specific laser light wavelength, verteporfin generates reactive oxygen species, causing targeted damage to the choroidal vasculature. Activated verteporfin also induces vasoconstriction and promotes choroidal vessel remodeling, facilitating SRF absorption and the decrease of choroidal hyperpermeability, while preserving the overlying retina. PDT involves the use of a laser light set at 689 nm with a fluence of 50 J/cm^2^ or 25 J/cm^2^, combined with an injection of verteporfin (Visudyne^®^ Novartis, Basel, Switzerland), to treat both acute and chronic CSCR [11,17,18].

The use of eplerenone oral therapy is based on the mineralocorticoid pathway hypothesis which suggests that, under normal physiological conditions, glucocorticoids occupy mineralocorticoid receptors (MR) in ocular tissues at basal levels [19]. However, MR activators such as aldosterone and corticosterone can lead to choroidal vessel dilation and leakage by upregulating the endothelial vasodilatory potassium channel KCa2.3. Dysregulation of MR by glucocorticoids contributes to choroidal vessel leakage and to serous retinal fluid accumulation observed in CSCR imaging [20]. Eplerenone, a MR antagonist initially developed to treat hypertension and heart failure, works by blocking MR also in the choroid. This action reduces vascular permeability and choroidal inflammation, potentially decreasing subretinal fluid accumulation. Clinical studies have demonstrated that eplerenone can improve subretinal fluid resolution and visual function in patients. Oral administration of eplerenone is generally well tolerated and has a favorable side effect profile compared to alternative therapies.

## 2. Materials and Methods

### 2.1. Study Design and Dataset

A retrospective analysis was performed on data collected from 53 eyes of 50 individuals with a confirmed diagnosis of persistent (≥6 months) simple or complex central serous chorioretinopathy (CSCR) treated at the Retina Service of the Azienda Ospedaliero-Universitaria delle Marche, Ancona, Italy, between September 2024 and March 2025 (Ancona IEC m. 13-219/2024—approved on 11 June 2024). Patients had originally been randomized in a 1:1 ratio using a computer-generated random sequence to receive either ICGA-guided half-dose full-fluence photodynamic therapy (HD-FF PDT) alone (Group A) or ICGA-guided HD-FF PDT combined with oral eplerenone (Group B). The present study represents a retrospective comparative analysis of these previously treated patients. In patients with bilateral involvement, both eyes were assigned to the same treatment group to avoid inter-treatment contamination. Given the limited number of bilateral cases, no adjustment for inter-eye correlation was performed. Allocation was concealed by centralized assignment based on a computer-generated randomization list. Due to the nature of the interventions, masking of patients and treating physicians was not feasible. However, OCT image evaluation and AI-based biomarker analysis were conducted independently of clinical data. The AI-based analysis was fully automated, and any manual corrections required were performed by trained observers masked to treatment allocation. HD-FF PDT was performed using verteporfin (3 mg/m^2^) with a full-fluence rate of 50 J/cm^2^, administered by a single experienced physician (ML). Verteporfin was infused over 10 min, followed by laser treatment 15 min after the start of the infusion. A laser light at 689 nm was applied at 50 J/cm^2^ with an intensity of 300 mW/cm^2^ for 83 s.

In Group B, oral eplerenone was started at least 2 weeks prior to photodynamic therapy and was continued throughout the 6-month follow-up period, unless treatment discontinuation was required for safety reasons. Eplerenone was administered at 25 mg/day for the first 7 days, and increased to 50 mg/day hereafter, provided serum potassium levels and renal function remained within the predefined safety thresholds. Treatment tolerance and adherence were monitored through periodic blood tests during follow-up. If potassium levels exceeded 4.5 mmol/L or if creatinine clearance fell below 60 mL/min, treatment was discontinued, and those patients were excluded from the study.

Functional data, including best-corrected visual acuity (BCVA), and morphological data, such as structural OCT, were collected at baseline, and at 1-, 3-, and 6-months following PDT.

All structural OCT scans were obtained using the Spectralis HRA + OCT2 platform (Heidelberg Engineering, Heidelberg, Germany). For each study eye, a volume scan of 20 × 20° (approximately 6 × 6 mm) consisting of 49 horizontal B-scans (automated real-time value set to 25 frames per scan) and a high-resolution horizontal B-scan (30° in length, automated real-time value set to 100 frames) were analyzed by an artificial intelligence (AI) algorithm.

The inbuilt Spectralis software (HEYEX 2.6.8) was used to evaluate the central macular thickness (CMT), defined as the distance between the internal limiting membrane (ILM) and Bruch’s membrane in the central foveal ring (1 mm diameter), according to the Early Treatment Diabetic Retinopathy Study (ETDRS) grid. The automatic grid placement and retinal segmentations were verified and manually corrected if necessary. Additionally, the software was used to measure the subfoveal choroidal thickness (SCT), conventionally assumed as the distance between the RPE and the choroidoscleral border [21]. The assessments were made manually by trained observers with the machine’s caliper.

To define a ‘complete SRF resolution,’ a cut-off of <0.010 mm^3^ was applied, while a cut-off >0.010 mm^3^ outlined a ‘partial SRF resolution.’ The distinction into complete or partial SRF resolution was conducted at the 6-month follow-up.

Exclusion criteria included: history or evidence of other chorioretinal diseases, such as age-related macular degeneration, diabetic retinopathy, epiretinal membranes, or macular dystrophy; history of intraocular surgery; evidence of glaucoma; poor image quality due to cataracts or unstable fixation. Subjects with systemic diseases or other conditions that could affect retinal or choroidal thickness were also excluded.

The study was conducted in accordance with the principles of the Declaration of Helsinki, and written informed consent was obtained from all patients prior to enrolment.

### 2.2. Al Algorithm Description and Analysis

**Figure 1:**
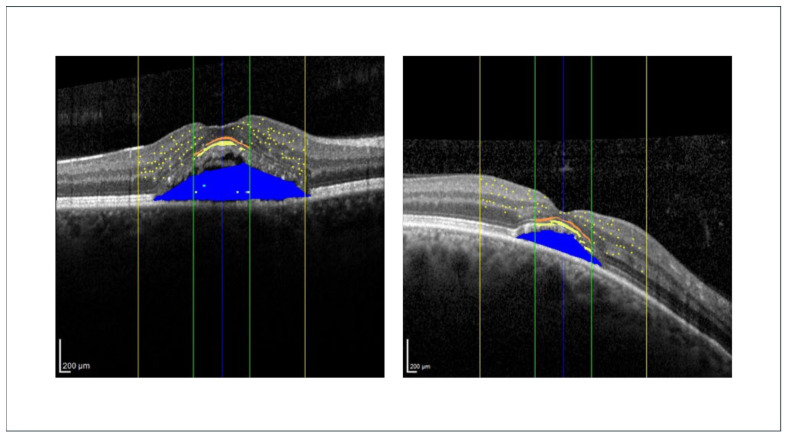
Summary of the distinct OCT biomarkers assessed using AI algorithm: subretinal fluid (blue); hyperreflective retinal foci (yellow dots) localized within the central 3 mm (yellow lines); external limiting membrane (orange) and ellipsoid zone (yellow) localized within the central 1 mm (green lines).

As previously described, our AI algorithm was based on adversarial generative networks, a deep learning technique [22,23]. The algorithm was trained and validated on a large dataset of retinal OCT images, including eyes with central serous chorioretinopathy, and its performance has been previously reported in the literature. Image quality thresholds were applied prior to analysis, and all AI outputs were generated independently of clinical data and treatment allocation; when required, minor segmentation corrections were performed by trained observers masked to group assignment. The AI algorithm allows the simultaneous evaluation of several OCT biomarkers, as illustrated in Figure 1. For each study eye, the AI algorithm was employed to analyze both volumetric and horizontal OCT scans. The collected data from the volumetric scan included the volumes of intraretinal fluid (IRF) and sub retinal fluid (SRF). The percentage of SRF volume (SRFV) within specific regions was assessed, such as the central 1 mm circle (SRF-1), the ring between 1 and 3 mm (SRF-3), and the region between 3 and 6 mm (SRF-6). Additionally, the AI algorithm evaluated the percentage of external limiting membrane (ELM) and ellipsoid zone (EZ) interruption within the central 1 mm of the central scan passing through the fovea. Moreover, the number of hyper-reflective foci (HRF) within the central 3 mm was computed in the high-resolution horizontal B-scan.

### 2.3. Retinal Biomarkers Evaluation

**Figure 2:**
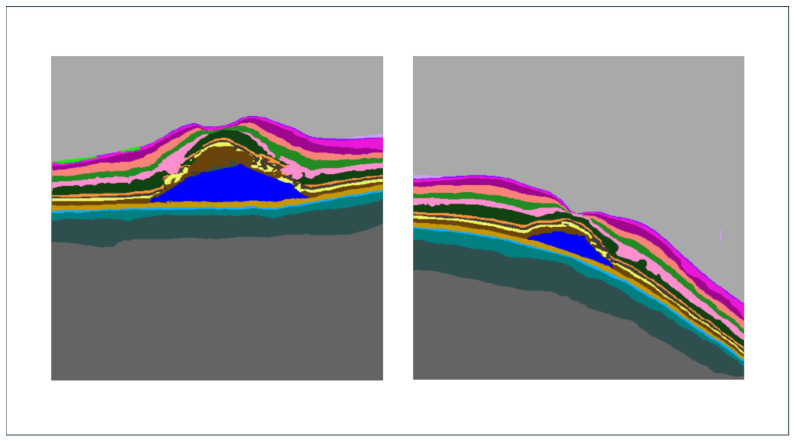
Automated retinal layer segmentation performed by the AI software. Subretinal fluid is highlighted in blue, while the remaining colors represent the segmented retinal layers.

The AI-based evaluation assessed the presence of IRF, SRF, ELM/EZ interruption and the number of HRF, as mentioned above. All these factors were computed at baseline, and 1-, 3-, 6-months after the PDT. For both volumetric and horizontal scans, we ensured the accuracy of the automated fovea centering and of the retinal layers segmentation (Figure 2). In case of segmentation inaccuracies, manual correction was performed by trained graders masked to treatment allocation.

### 2.4. Statistical Analyses

Quantitative variables are presented as means and standard deviations (SD), while qualitative variables are presented with percentage tables and/or graphs. The Shapiro-Wilk test was used to assess whether the data followed a normal distribution.

The longitudinal analysis, based on the results of the normality test, used either repeated measures ANOVA or the Friedman rank test. Post-hoc analysis was performed using the Bonferroni test. The comparison between independent groups (PDT = 0, PDT + EPL = 1) was carried out using either the *t*-test or the Wilcoxon-Mann-Whitney test.

Qualitative variables were evaluated using the chi-square test. Statistical significance was set at *p* < 0.05. All calculations were performed using IBM SPSS Software V. 25.0.0.

## 3. Results

### 3.1. Baseline Data

Our cohort consisted of 53 eyes of 50 patients, 46 men (87%) and 7 women (13%), with a mean age of 52 years. Group A included 27 eyes of 25 patients, with a mean age of 53.7 ± 8.9 years, while Group B included 26 eyes from 25 patients, with a mean age of 51.1 ± 10.2 years (*p* = 0.306). Three patients had bilateral CSCR involvement, and both eyes were therefore assigned to the same treatment group and included in the analysis. Given the limited number of bilateral cases, no adjustment for inter-eye correlation was deemed necessary. No patients discontinued eplerenone or were excluded after randomization due to safety concerns. The study population was mildly hyperopic, with a mean spherical equivalent of +1.50 diopters, and most eyes (81%) exhibited flat irregular pigment epithelial detachment (FIPED), defined as a shallow, horizontally oriented and irregular elevation of the retinal pigment epithelium on structural OCT, as previously described in pachychoroid spectrum diseases on structural OCT at enrolment [24,25].

Mean baseline BCVA was 0.32 log-MAR in Group A and 0.25 log-MAR in Group B (*p* = 0.098). Mean baseline subretinal fluid volume was 0.325 mm^3^ ± 0.385 in Group A and 0.329 mm^3^ ± 0.366 in Group B (*p* = 0.965). In addition, no statistically significant differences were observed at baseline for the other analyzed parameters, such as intraretinal fluid volume (IRFV), HRF, and EZ and ELM percentage of interruption within 1 mm from the central fovea (Table 1). Regarding the mean CMT and SCT at baseline, in Group A the values were 299.7 µm ± 73 µm and 447.5 µm ± 53.2 µm, respectively. While in Group B, the mean CMT was 344 µm ± 87.2 µm (*p* = 0.051) and the mean SCT was 453.9 µm ± 58.4 µm (*p* = 0.68).

### 3.2. Functional Outcomes Data

At the 1-month follow-up, visual function showed an improvement in both groups. In Group A, mean BCVA moved from 0.32 ± 0.16 to 0.26 ± 0.18 log-MAR, while in Group B the improvement was greater with a mean BCVA which moved from 0.25 ± 0.14 to 0.16 ± 0.14 log-MAR (*p* = 0.032). This amelioration was maintained in both groups also at the 3- and 6-months follow-up. At 3 months, the mean BCVA was 0.22 ± 0.21 log-MAR in Group A and 0.13 ± 0.10 log-MAR in Group B, reflecting a mean improvement of 0.038 log-MAR from the previous visit (*p* = 0.051). No statistically significant difference was observed between the two groups at 3 months. At 6 months, the mean BCVA was found to be better in Group B compared to Group A, with a value of 0.1 ± 0.12 log-MAR in the former and 0.21 ± 0.18 in the latter (*p* = 0.009). Additionally, the mean BCVA at 6 months compared to baseline improved from 0.32 ± 0.16 to 0.21 ± 0.18 log-MAR in Group A and from 0.25 ± 0.14 to 0.1 ± 0.12 log-MAR in Group B (Table 2).

### 3.3. Retinal Biomarkers Data

**Figure 3:**
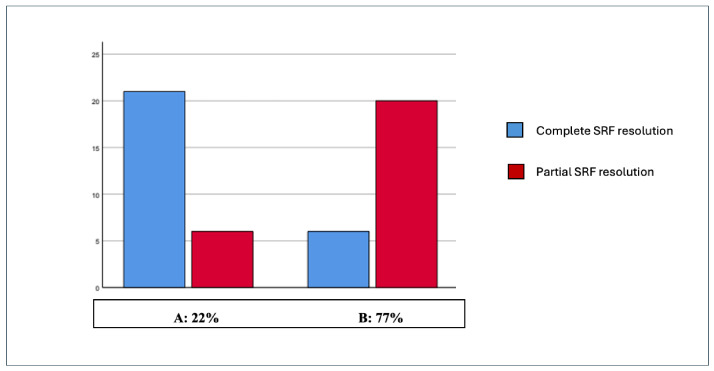
On the left-side Group A, on the right-side Group B. Complete SRF resolution (SRF < 0.010 mm^3^) represented in blue, partial SRF resolution (SRF > 0.010 mm^3^) represented in red.

At baseline, the mean SRFV was 0.325 mm^3^ ± 0.385 mm^3^ in Group A and 0.329 ± 0.366 mm^3^ in Group B (*p* = 0.965). A reduction was observed in both groups at all follow-up visits. Specifically, in Group A the mean SRFV decreased from 0.325 mm^3^ to 0.216 mm^3^ at 1-month post-treatment, 0.214 mm^3^ at 3 months, and 0.199 mm^3^ at 6 months. In Group B the mean SRFV decreased from 0.329 mm^3^ at baseline to 0.158 mm^3^ at one-month post-treatment, 0.081 mm^3^ at 3 months, and 0.021 mm^3^ at 6 months. At the 6-months follow-up, Group B showed a statistically significant reduction in SRFV compared to Group A (*p* = 0.014) (Table 3). Furthermore, considering a SRFV lower than 0.010 mm^3^ as a complete SRF resolution, we observed that 77% of patients in Group B experienced a complete resolution of SRF, while in Group A this occurred in 22% of patients (*p* = 0.001) (Figure 3).

Among the parameters analyzed, aside from SRFV, no statistically significant difference was observed at any follow-up visits. The biomarkers considered were: IRFV, HRF, percentage of ELM and EZ interruption within the central 1 mm from the fovea.

#### Central Macular Thickness and Sub-Foveal Choroidal Thickness

The mean CMT decreased in both groups, although there was no statistically significant difference. In Group A, the CMT was 297 ± 73 µm at baseline and 236 ± 87 µm at 6-months, reflecting an average reduction in macular thickness of 61 µm. In Group B, the baseline CMT was 344 ± 87 µm and at 6 months the CMT reduced to 221 ± 55 µm, showing an average difference of 124 µm (*p* = 0.46) (Table 4). The mean SCT showed no statistically significant difference at baseline (Group A 447.5 ± 53.2 µm, Group B 453.9 µm ± 58.5; *p* = 0.068). At the 6-months follow-up, the mean SCT decreased in both groups. In Group A the SCT reached 416.7 µm ± 44.5 at 6 months, with a decrease of 30.8 µm compared to baseline, while in Group B the SCT was 422.1 µm ± 55.6, with an average reduction of 31.8 µm. No substantial statistically significant difference was found between the two groups at any follow-up visits (Table 5).

## 4. Discussion

In recent years, the debate surrounding the use of MR inhibitors in the treatment of acute/chronic CSCR has become increasingly heated, bringing this topic to the forefront. The aim of this study was to assess whether the combination of eplerenone and HD-PDT provides greater efficacy compared to HD-PDT monotherapy in treating persistent CSCR.

PDT is widely regarded as the “gold standard” treatment for chronic CSCR and several randomized controlled trials (RCTs) have provided evidences supporting it [26]. HD-PDT has been shown to be as effective as, or even superior to, full-dose, half-fluence, and half-time PDT regimens in treating CSCR [27]. Furthermore, a reduced verteporfin dose seems to have a better safety profile and similar efficacy compared to a full-dose approach. In parallel with PDT, other therapeutic approaches have been explored in the attempt to optimize anatomical outcomes in CSCR. In recent years, alternative treatment strategies for CSCR, including subthreshold micropulse laser (SML), have been proposed and compared with both photodynamic therapy and mineralocorticoid receptor antagonists. Comparative studies have shown that while SML may achieve anatomical improvement in selected cases, ICGA-guided PDT generally provides faster and more consistent subretinal fluid resolution, particularly in chronic or persistent CSCR. Toto et al. reported superior anatomical outcomes with PDT compared to SML, especially in terms of SRF resolution and choroidal remodeling [28]. Similarly, Ruggeri et al. highlighted that MR antagonist therapy and SML may play a role in selected patients, but PDT remains the most effective approach for achieving sustained anatomical control in chronic disease [29]. These findings further support the rationale for exploring combined therapeutic strategies, as investigated in the present study. Nevertheless, not all cases respond with complete resolution after PDT. Hence the need to find an adjuvant therapy with a good safety profile such as oral eplerenone. Unfortunately, the two main RCTs focusing on oral eplerenone treatment for CSCR (VICI and SPECTRA trials) did not provide strong evidence regarding the therapeutic prospects of this drug [30,31]. On the other hand, many small, non-randomized, retrospective studies have shown potentially favorable outcomes following oral eplerenone treatment for CSCR, particularly in terms of subretinal fluid reduction and visual acuity improvement [32,33,34].

What makes our study unique is the comparison between PDT as monotherapy and its combination with oral eplerenone, whereas other studies focused on eplerenone as monotherapy. For this reason, in our discussion we can only relate our findings and refer to other studies in a manner that is not entirely exhaustive. This topic is scarcely explored in literature to date, which has sparked significant interest for us.

In our study, it emerged that both groups showed a substantial improvement in all the parameters examined. This is significant in terms of the efficacy of the largely proposed treatment, reaffirming that PDT is an effective treatment for CSCR [19].

Considering BCVA, it improved by approximately one line of Log-MAR in both groups just one month after treatment. Furthermore, the visual acuity showed a greater improvement in the combined therapy group, with a statistically significant difference from baseline at both 1-month (*p* = 0.032) and 6-months (*p* = 0.009) follow-up. Our results are consistent with the findings of Rahimy et al., who conducted a prospective, randomized, double-blind, placebo-controlled study in which patients had a confirmed diagnosis of CSCR for at least 3 months [35]. In their research, BCVA improved from 0.394 to 0.330 Log-MAR (*p* = 0.04) after 9 weeks of eplerenone therapy, while the placebo group BCVA slightly decreased from 0.313 to 0.342 Log-MAR during the same period (*p* = 0.21). Also Rajesh et al. found in a prospective non-randomized study a mean BCVA improvement from 0.27 to 0.19 Log-MAR in patient with chronic CSCR treated with oral eplerenone [36]. Other authors have demonstrated as well that intervening on the cascade of the pathway regulated by MRs can have beneficial effects in improving the course of the disease and, consequently, visual acuity, as shown by Herold et al. in their interventional uncontrolled open-label prospective clinical trial in patient affected by non-resolving CSCR. In their study, 47 eyes of 21 patients were treated with 25 mg of spironolactone twice daily for 3 months and the mean BCVA improved from 0.25 to 0.17 Log-MAR [37].

Considering the structural parameters, particularly the SRFV, our results reveal that patients treated with combined therapy experienced a greater and more lasting resorption of subretinal fluid compared to the group that received PDT alone. After one month, the mean SRFV in the combined therapy group nearly halved, decreasing from an average value of 0.329 mm^3^ to 0.158 mm^3^, and ultimately reaching a mean volume of 0.021 mm^3^ with a standard deviation of only ±0.052 mm^3^ after 6 months. This data suggests that patients who underwent continuous eplerenone therapy, even after PDT, may be less prone to recurrences and may maintain a dry retina for a longer duration. In our sample, at the 6 months follow-up, the amount of SRF was significantly lower in Group B compared to Group A. However, the SRFV decreased substantially in both groups, suggesting that PDT plays a major role in the resolution of exudative phenomena. Nevertheless, Bousquet et al. showed a significant decreased (*p* < 0.01) in SRF and a significant improvement (*p* < 0.001) in BCVA in patient with persistent CSCR (>4 months) treated with oral eplerenone versus placebo [32]. Another parameter considered by Bousquet et al. was the CMT, which decreased significantly from 352 ± 139 μm at baseline to 246 ± 113 μm and 189 ± 99 μm at 1 and 3 months under eplerenone treatment (*p* < 0.05 and *p* < 0.01, respectively) [32]. In our cohort, both groups experienced a significant reduction in CMT. In Group A, the baseline CMT was 297 µm ± 73 µm, and at the 6-month follow-up, it reached 236 µm ± 87 µm, reflecting an average reduction of 61 µm. In Group B, the baseline CMT was 344 µm ± 87 µm, and at 6 months, it reduced to 221 µm ± 55 µm, showing an average difference of 124 µm. Although this data does not reach statistical significance, it is undoubtedly clear that in the group of patients who received combined therapy there was a twofold greater reduction in CMT. However, at 6 months, the average CMT in Group B is lower than in Group A, and this is certainly an important data to consider.

Regarding SCT, there was no statistically significant difference between the two groups at any of the follow-up visits. Nevertheless, we observed a gradual, progressive, and balanced thinning of the choroidal thickness of about 30 µm in both groups. We know that choroidal thickness may temporarily increase following HD-PDT, and this transient choroidal thickening can be accompanied by a temporary increase in SRF [38]. These treatment-related changes generally last about 1–3 weeks after PDT, with a gradual improvement in both anatomical and functional outcomes. Our outcomes align with those described by Maruko et al., who reported that HD-PDT for CSCR led to a thinner SCT already at 1 month and this trend was maintained at 3, 6, and 12 months after treatment [38].

Among the other retinal biomarkers analyzed, no statistically significant differences were observed at any of the follow-up visits conducted at 1-, 3-, and 6-months.

The last point we would like to address concerns the definition of complete resolution of SRF. As previously mentioned, we used an AI algorithm to measure SRF, and in some cases we encountered a situation where the SRF no longer appeared clinically significant, nor could it be objectively detected through the observation of the structural OCT scans. Despite this, the software still recognized some fluid under the retina. Therefore, in agreement, our study group proposed to define the complete resolution of SRF as a SRFV inferior to 0.010 mm^3^. In this way, at the 6-months follow-up, a complete SRF resolution was gained in the 22% of patients in Group A and in the 77% of patients in Group B. Similarly, in a prospective interventional case-control study conducted on 58 patients with acute CSCR, Vankatesh et al. reported a complete SRF resolution in 62% patient treated with oral eplerenone at 3 months and, in the observation group, complete SRF resolution was noted in just 31% at the same time (*p* < 0.001) [33]. In a retrospective study of 110 eyes of 83 patients with cCSC, Petkovsek et al. found that one year after eplerenone treatment 33% of eyes had complete SRF resolution [39]. In the PLACE trial, Van Dijk et al. reported a SRF resolution of 51% at 6–8 weeks and of 67% at 7–8 months following ICGA-guided HD-PDT in cCSCR [40].

## 5. Conclusions

While PDT remains the gold standard treatment for persistent CSCR, we hypothesize that combined therapy may have a synergistic effect on SRF resorption. This could represent a therapeutic strategy targeting two distinct pathways: the choroidal vessel remodeling through PDT and the downregulation of the overactivated MR pathway in choroidal vessels via eplerenone. Clinically, this could translate into an accelerated SRF resorption rate reducing the risk of potential recurrences.

Although there is no evidence in literature supported by RCTs regarding the effectiveness of eplerenone in the treatment of CSCR, in our cohort the addition of a MR antagonist proved to be effective both functionally and structurally, improving the BCVA of our patients and influencing the amount of SRFV. Furthermore, it is essential to focus on additional therapeutic strategies to address the treatment of CSCR. RCTs are required to evaluate the effectiveness and the optimal administration methods of MR inhibitors in the treatment of CSCR.

## Figures and Tables

**Table 1:** Baseline demographic and clinical characteristics of the study groups. Baseline values for patients in Group A (HD-FF PDT) and Group B (HD-FF PDT plus oral eplerenone). Continuous variables are presented as mean ± standard deviation (SD). *p* values refer to between-group comparisons at baseline.

	Group A (PDT)		Group B (PDT + Eplerenone)		
	Mean	SD	Mean	SD	*p*-Value
Age	53.74	±8.2	51.12	±10.1	0.306
BCVA log-MAR	0.32	±0.2	0.25	±0.1	0.098
SRFV mm^3^	0.32	±0.4	0.32	±0.4	0.965
IRFV mm^3^	0.002	±0.003	0.004	±0.006	0.09
SCT µm	447.5	±53.2	453.9	±58.4	0.68
CMT µm	297	±73	344	±87.2	0.051
HRF	83.9	±26.6	90.2	±29.7	0.421

Abbreviations: BCVA = best-corrected visual acuity, CMT = central macular thickness, SCT = subfoveal choroidal thickness, SRFV = subretinal fluid volume, IRFV = intraretinal fluid volume, HRF = hyperreflective foci, PDT = photodynamic therapy, SD = standard deviation.

**Table 2:** Visual acuity outcomes. Mean BCVA at baseline and follow-up visits in Group A (PDT) and Group B (PDT + eplerenone). Data are reported as mean ± SD; *p* values refer to between-group comparisons.

	Group A (PDT)		Group B (PDT + Eplerenone)		
Log-MAR	Mean	SD	Mean	SD	*p*-Value
BCVA 0	0.32	±0.16	0.25	±0.14	0.098
BCVA 1	0.26	±0.18	0.16	±0.14	0.032
BCVA 3	0.22	±0.21	0.13	±0.10	0.051
BCVA 6	0.21	±0.18	0.1	±0.12	0.009

Abbreviations: BCVA = best-corrected visual acuity, PDT = photodynamic therapy, SD = standard deviation.

**Table 3:** Subretinal fluid volume. Mean SRFV at baseline and follow-up visits in Group A (PDT) and Group B (PDT + eplerenone). Data are reported as mean ± SD; *p* values refer to between-group comparisons.

	Group A (PDT)		Group B (PDT + Eplerenone)		
mm^3^	Mean	SD	Mean	SD	*p*-Value
SRFV 0	0.325	±0.385	0.329	±0.366	0.965
SRFV 1	0.216	±0.319	0.158	±0.356	0.540
SRFV 3	0.214	±0.348	0.081	±0.14	0.075
SRFV 6	0.199	±0.354	0.021	±0.052	0.014

Abbreviations: PDT = photodynamic therapy, SRFV = subretinal fluid volume, SD = standard deviation.

**Table 4:** Central macular thickness. Mean CMT at baseline and follow-up visits in Group A (PDT) and Group B (PDT + eplerenone). Data are reported as mean ± SD; *p* values refer to between-group comparisons.

	Group A (PDT)		Group B (PDT + Eplerenone)		
µm	Mean	SD	Mean	SD	*p*-Value
CMT 0	299.7	±73	344	±87.2	0.038
CMT 1	256.5	±71.4	270.4	±90.4	0.535
CMT 3	238.9	±78.4	253.3	±81	0.512
CMT 6	235.8	±87.5	220.8	±55.3	0.46

Abbreviations: PDT = photodynamic therapy, CMT = central macular thickness, SD = standard deviation.

**Table 5:** Subfoveal choroidal thickness. Mean SCT at baseline and follow-up visits in Group A (PDT) and Group B (PDT + eplerenone). Data are reported as mean ± SD; *p* values refer to between-group.

	Group A (PDT)		Group B (PDT + Eplerenone)		
µm	Mean	SD	Mean	SD	*p*-Value
SCT 0	447.5	±53.2	453.9	±58.5	0.068
SCT 1	425.96	±56.1	432.1	±57.7	0.698
SCT 3	420.7	±40.9	422.4	±49.8	0.891
SCT 6	416.7	±44.5	422.1	±55.6	0.699

Abbreviations: SCT = subfoveal choroidal thickness, PDT = photodynamic therapy, SD = standard deviation.

## Data Availability

The data supporting the findings of this study are available from the corresponding author upon reasonable request.

## Abbreviations

The following abbreviations are used in this manuscript:

AI	Artificial Intelligence
AMD	Age-Related Macular Degeneration
BCVA	Best-Corrected Visual Acuity
CMT	Central Macular Thickness
CSCR	Central Serous Chorioretinopathy
cCSCR	Chronic Central Serous Chorioretinopathy
DR	Diabetic Retinopathy
ELM	External Limiting Membrane
ETDRS	Early Treatment Diabetic Retinopathy Study
EZ	Ellipsoid Zone
FIPED	Flat Irregular Pigment Epithelial Detachment
HD-FF PDT	Half-Dose Full-Fluence Photodynamic Therapy
HD-PDT	Half-Dose Photodynamic Therapy
HRF	Hyperreflective Foci
ICGA	Indocyanine Green Angiography
IEC	Institutional Ethics Committee
ILM	Internal Limiting Membrane
IRF	Intraretinal Fluid
IRFV	Intraretinal Fluid Volume
logMAR	Logarithm of the Minimum Angle of Resolution
MR	Mineralocorticoid Receptor
OCT	Optical Coherence Tomography
PDT	Photodynamic Therapy
RCT	Randomized Controlled Trial
RPE	Retinal Pigment Epithelium
RVO	Retinal Vein Occlusion
SD	Standard Deviation
SCT	Subfoveal Choroidal Thickness
SML	Subthreshold Micropulse Laser
SRF	Subretinal Fluid
SRFV	Subretinal Fluid Volume
VEGF	Vascular Endothelial Growth Factor

## References

[1] Chhablani J. (2019). Central Serous Chorioretinopathy.

[2] Reiner A., Fitzgerald M.E.C., Del Mar N., Li C. (2018). Neural Control of Choroidal Blood Flow. Prog. Retin. Eye Res..

[3] Jain M., Mohankumar A., Torres J., Alkorbi H.A. (2025). Pharmacological Associations of Central Serous Chorioretinopathy: Looking beyond Corticosteroids. Surv. Ophthalmol..

[4] Ben-Eli H., Asher T., Lender R., Mirsky D., Abu-Shkara R., Hamuda M., Aslee N., Marei H., Flug R., Eitan R. (2025). Anterior Segment Characteristics and Quality of Life of Patients with Central Serous Chorioretinopathy. J. Clin. Med..

[5] Brown R.B., Mohan S., Chhablani J. (2023). Pachychoroid Spectrum Disorders: An Updated Review. J. Ophthalmic Vis. Res..

[6] Frederiksen I.N., Arnold-Vangsted A., Anguita R., Boberg-Ans L.C., Cehofski L.J., van Dijk E.H.C., Eriksen N.S., Ferro Desideri L., Grauslund J., Huemer J. (2025). Global Incidence of Central Serous Chorioretinopathy: A Systematic Review, Meta-Analysis, and Forecasting Study. Ophthalmol. Ther..

[7] Rabiolo A., Zucchiatti I., Marchese A., Baldin G., Sacconi R., Montorio D., Cicinelli M.V., Querques L., Bandello F., Querques G. (2018). Multimodal Retinal Imaging in Central Serous Chorioretinopathy Treated with Oral Eplerenone or Photodynamic Therapy. Eye.

[8] Cardillo Piccolino F., Lupidi M., Cagini C., Fruttini D., Nicolò M., Eandi C.M., Tito S. (2018). Retinal Vascular Reactivity in Central Serous Chorioretinopathy. Investig. Ophthalmol. Vis. Sci..

[9] Piccolino F.C., Fruttini D., Eandi C., Nicolò M., Mariotti C., Tito S., Lupidi M. (2022). Vigorous Physical Activity as a Risk Factor for Central Serous Chorioretinopathy. Am. J. Ophthalmol..

[10] Meduri A., Merlo E.M., Sparacino G., Mancini M., Oliverio G.W., Silvestro O., De Luca L., Martino G., Aragona P. (2025). Beyond the Vision: Central Serous Chorioretinopathy, Anxiety and Depression-a Systematic Review. Front. Med..

[11] Feenstra H.M.A., van Dijk E.H.C., Cheung C.M.G., Ohno-Matsui K., Lai T.Y.Y., Koizumi H., Larsen M., Querques G., Downes S.M., Yzer S. (2024). Central Serous Chorioretinopathy: An Evidence-Based Treatment Guideline. Prog. Retin. Eye Res..

[12] Chhablani J., Behar-Cohen F., Central Serous Chorioretinopathy International Group (2022). Validation of Central Serous Chorioretinopathy Multimodal Imaging-Based Classification System. Graefes Arch. Clin. Exp. Ophthalmol..

[13] Singh S.R., Matet A., van Dijk E.H.C., Daruich A., Fauser S., Yzer S., Peiretti E., Sivaprasad S., Lotery A.J., Boon C.J.F. (2019). Discrepancy in Current Central Serous Chorioretinopathy Classification. Br. J. Ophthalmol..

[14] Radke N.V., van Dijk E.H.C., Spaide R.F., Holz F.G., Koizumi H., Freund K.B., Subhi Y., Lange C., Singh S.R., Chen H. (2025). International Consensuses and Guidelines on Central Serous Chorioretinopathy (CSC) by the Asia Pacific Vitreo-Retina Society (APVRS), the Academy of Asia-Pacific Professors of Ophthalmology (AAPPO) and the Academia Retina Internationalis (ARI). Asia Pac. J. Ophthalmol..

[15] Fung A.T., Yang Y., Kam A.W. (2023). Central Serous Chorioretinopathy: A Review. Clin. Exp. Ophthalmol..

[16] van Rijssen T.J., Mohabati D., Dijkman G., Theelen T., de Jong E.K., van Dijk E.H.C., Boon C.J.F. (2018). Correlation between Redefined Optical Coherence Tomography Parameters and Best-Corrected Visual Acuity in Non-Resolving Central Serous Chorioretinopathy Treated with Half-Dose Photodynamic Therapy. PLoS ONE.

[17] Fujita K., Imamura Y., Shinoda K., Matsumoto C.S., Mizutani Y., Hashizume K., Mizota A., Yuzawa M. (2015). One-Year Outcomes with Half-Dose Verteporfin Photodynamic Therapy for Chronic Central Serous Chorioretinopathy. Ophthalmology.

[18] Zhang X., Lim C.Z.F., Chhablani J., Wong Y.M. (2023). Central Serous Chorioretinopathy: Updates in the Pathogenesis, Diagnosis and Therapeutic Strategies. Eye Vis..

[19] Hirata M., Tsujikawa A., Matsumoto A., Hangai M., Ooto S., Yamashiro K., Akiba M., Yoshimura N. (2011). Macular Choroidal Thickness and Volume in Normal Subjects Measured by Swept-Source Optical Coherence Tomography. Investig. Ophthalmol. Vis. Sci..

[20] Leclercq B., Weiner A., Zola M., Mejlacowicz D., Lassiaz P., Jonet L., Gélizé E., Perrot J., Viengchareun S., Zhao M. (2023). The Choroidal Nervous System: A Link between Mineralocorticoid Receptor and Pachychoroid. Acta Neuropathol..

[21] Chhablani J., Anantharaman G., Behar-Cohen F., Boon C., Manayath G., Singh R. (2018). Management of Central Serous Chorioretinopathy: Expert Panel Discussion. Indian J. Ophthalmol..

[22] Jiang T., Gradus J.L., Rosellini A.J. (2020). Supervised Machine Learning: A Brief Primer. Behav. Ther..

[23] Midena E., Toto L., Frizziero L., Covello G., Torresin T., Midena G., Danieli L., Pilotto E., Figus M., Mariotti C. (2023). Validation of an Automated Artificial Intelligence Algorithm for the Quantification of Major OCT Parameters in Diabetic Macular Edema. J. Clin. Med..

[24] Bousquet E., Bonnin S., Mrejen S., Krivosic V., Tadayoni R., Gaudric A. (2018). Optical coherence tomography angiography of flat irregular pigment epithelium detachment in chronic central serous chorioretinopathy. Retina.

[25] Quarta A., Feo A., Corradetti G., Popovic M.M., Sadda S.R. (2025). Double Layer Sign in Chorioretinal Diseases: Clinical Significance and Implications from Multimodal Imaging. Surv. Ophthalmol..

[26] Kim Y.J., Sivaprasad S., Aslam T., Jaki Mekjavić P., Balčiūnienė V.J., Visser L., Joussen A.M., Yoon Y.H., Lai T.Y.Y., Okada A.A. (2025). Treatment of Central Serous Chorioretinopathy: New Options for an Old Disease. Eye.

[27] Liu C.-F., Chen L.-J., Tsai S.H., Lai C.-C., Chan W.-C., Wu W.-C., Wang N.-K., Chen K.-J., Hwang Y.-S., Chen Y.-P. (2014). Half-Dose Verteporfin Combined with Half-Fluence Photodynamic Therapy for Chronic Central Serous Chorioretinopathy. J. Ocul. Pharmacol. Ther..

[28] Toto L., Ares I., Quarta A., Viggiano P., Ruggeri M., Formenti F., Boscia G., Porreca A., Di Nicola M., Boscia F. (2025). Visual and Anatomical Evaluation of Navigated Subthreshold Micropulse Laser versus Photodynamic Therapy in Managing Chronic Central Serous Chorioretinopathy. Graefes Arch. Clin. Exp. Ophthalmol..

[29] Ruggeri M.L., Di Nicola M., Passamonti M., Lorenzi C., Quarta A., Mastropasqua R., Toto L. (2024). Choroidal and Choriocapillaris Changes after Photodynamic Therapy and Subthreshold Micropulse Laser Treatment for Central Serous Chorioretinopathy. Medicina.

[30] Lotery A., Sivaprasad S., O’Connell A., Harris R.A., Culliford L., Cree A., Madhusudhan S., Griffiths H., Ellis L., Chakravarthy U. (2021). Eplerenone Versus Placebo for Chronic Central Serous Chorioretinopathy: The VICI RCT.

[31] van Rijssen T.J., van Dijk E.H.C., Tsonaka R., Feenstra H.M.A., Dijkman G., Peters P.J.H., Diederen R.M.H., Hoyng C.B., Schlingemann R.O., Boon C.J.F. (2022). Half-Dose Photodynamic Therapy Versus Eplerenone in Chronic Central Serous Chorioretinopathy (SPECTRA): A Randomized Controlled Trial. Am. J. Ophthalmol..

[32] Bousquet E., Beydoun T., Zhao M., Hassan L., Offret O., Behar-Cohen F. (2013). Mineralocorticoid Receptor Antagonism in the Treatment of Chronic Central Serous Chorioretinopathy: A Pilot Study. Retina.

[33] Venkatesh R., Pereira A., Jayadev C., Prabhu V., Aseem A., Jain K., Bavaharan B., Yadav N.K., Chhablani J. (2020). Oral Eplerenone Versus Observation in the Management of Acute Central Serous Chorioretinopathy: A Prospective, Randomized Comparative Study. Pharmaceuticals.

[34] Han J.Y., Kim Y.J., Choi E.Y., Lee J., Lee J.H., Kim M., Byeon S.H., Kim S.S., Lee C.S. (2022). Therapeutic Efficacy of Spironolactone for Central Serous Chorioretinopathy. Yonsei Med. J..

[35] Rahimy E., Pitcher J.D., Hsu J., Adam M.K., Shahlaee A., Samara W.A., Vander J.F., Kaiser R.S., Chiang A., Spirn M.J. (2018). A randomized double-blind placebo-control pilot study of eplerenone for the treatment of central serous chorioretinopathy (ecselsior). Retina.

[36] Rajesh B., Agrawal H., Peguda H.K., Chhablani J. (2018). Predictors of Outcome During Eplerenone Therapy in Chronic Central Serous Chorioretinopathy:A Prospective, Open-Label Pilot Clinical Study. Ophthalmic Surg. Lasers Imaging Retin..

[37] Herold T.R., Rist K., Priglinger S.G., Ulbig M.W., Wolf A. (2017). Long-Term Results and Recurrence Rates after Spironolactone Treatment in Non-Resolving Central Serous Chorio-Retinopathy (CSCR). Graefes Arch. Clin. Exp. Ophthalmol..

[38] Maruko I., Iida T., Sugano Y., Ojima A., Ogasawara M., Spaide R.F. (2010). Subfoveal Choroidal Thickness after Treatment of Central Serous Chorioretinopathy. Ophthalmology.

[39] Petkovsek D.S., Cherfan D.G., Conti F.F., Hom G.L., Ehlers J.P., Babiuch A.S., Rachitskaya A.V., Kaiser P.K., Schachat A.P., Srivastava S.K. (2020). Eplerenone for the Treatment of Chronic Central Serous Chorioretinopathy: 3-Year Clinical Experience. Br. J. Ophthalmol..

[40] van Dijk E.H.C., Fauser S., Breukink M.B., Blanco-Garavito R., Groenewoud J.M.M., Keunen J.E.E., Peters P.J.H., Dijkman G., Souied E.H., MacLaren R.E. (2018). Half-Dose Photodynamic Therapy versus High-Density Subthreshold Micropulse Laser Treatment in Patients with Chronic Central Serous Chorioretinopathy: The PLACE Trial. Ophthalmology.

